# *Mycobacterium mageritense* Lymphadenitis in Child

**DOI:** 10.3201/eid2803.211486

**Published:** 2022-03

**Authors:** Miguel García-Boyano, Fernando Baquero-Artigao, Carlos Toro, Marina Alguacil-Guillén, Fernando Lázaro-Perona, Cristina Calvo

**Affiliations:** Hospital Universitario La Paz, Madrid, Spain (M. García-Boyano, F. Baquero-Artigao, C. Toro, M. Alguacil-Guillén, A. Lázaro-Perona, C. Calvo);; Translational Research Network of Pediatric Infectious Diseases, Madrid (F. Baquero-Artigao, C. Calvo);; CIBERINFEC, Instituto de Salud Carlos III, Madrid (F. Baquero-Artigao, C. Calvo)

**Keywords:** Mycobacterium mageritense, bacteria, lymphadenitis, respiratory infections, tuberculosis and other mycobacteria, child, antimicrobial resistance, Spain

## Abstract

Although human infections caused by *Mycobacterium mageritense* are rare, there are some case reports involving sinusitis, pneumonia, and hospital-acquired infections in adults. We report a case of lymphadenitis caused by *M. mageritense* in a child in Spain.

*Mycobacterium mageritense* was identified as a novel distinct species in 1997. Its name is derived from Magerit, the old Arabic name of Madrid, Spain, the source of most of the human sputum specimens from which it was first isolated (*1*). Five years later, cases of clinical disease caused by *M. mageritense* were reported in adults (*2*). We report a case of lymphadenitis caused by *M. mageritense* in a child in Spain.

A previously healthy boy, 2 years and 9 months of age, came to a pediatric clinic because of a 1-week history of swelling of the right submandibular lymph node. Physical examination showed lymph node swelling in the right submandibular region with red‒violet discolored skin. He did not had a fever, pain, or any other signs and symptoms. An ultrasound examination showed an enlarged submandibular lymph node 18 mm in diameter. Laboratory studies showed a leukocyte count of 9,220 cells/mm^3^ (reference range 4,800‒15,000 cells/mm^3^), a differential count of 42% neutrophils (reference range 55%‒70%), and a C-reactive protein level of <0.05 mg/dL (reference range <1‒0.5 mg/dL).

Three days later, he underwent fine-needle aspiration of the involved lymph node. Histopathologic analysis showed necrotizing granulomatous lymphadenitis. Acid-fast bacillus staining was negative. Therefore, a nontuberculous mycobacterial lymphadenitis was suspected and treatment with oral clarithromycin (7.5 mg/kg every 12 h) and ciprofloxacin (15 mg/kg every 12 h) was started.

A rapidly growing mycobacterium was isolated from the lymph node specimen after 6 days of incubation in liquid culture medium (BBL Mycobacteria Growth Indicator Tube; Becton Dickinson, https://www.bd.com). It was identified as *M. mageritense* by using GenoType Mycobacterium CM version 2.0 (Hain Lifescience, https://www.hain-lifescience.de). Matrix-assisted laser desorption/ionization time-of-flight mass spectrometry (Bruker Daltonics, https://www.bruker.com) yielded a score of 2.4 for *M. mageritense*. Whole-genome sequencing was performed to confirm these findings (GenBank accession no. JAJJNE010000000).

Susceptibility testing using a microdilution technique showed a susceptible MIC for linezolid (8 μg/mL); an intermediate MIC for moxifloxacin (2 μg/mL), imipenem (8 μg/mL), and cefoxitin (32 μg/mL); and a resistant MIC for trimethoprim/sulfamethoxazole (>8/152 μg/mL), ciprofloxacin (>4 μg/mL), amikacin (>64 μg/mL), clarithromycin (>16 μg/mL), doxycycline (>16 μg/mL), and tobramycin (>16 μg/mL). Breakpoints were those suggested by the Clinical and Laboratory Standards Institute for rapidly growing mycobacteria (*3*).

Accordingly, 3 weeks after fine-needle aspiration was performed, clarithromycin was replaced by oral linezolid (10 mg/kg every 8 h). However, this change was promptly stopped because of intolerance to linezolid, and clarithromycin was given again. The enlarged lymph node gradually improved, and antimicrobial drug treatment was discontinued 11 weeks after initial prescription. The lymph node was reduced to <50% of its initial size. Complete excision of residual lymph node and scar tissue was performed 2 months later, leading to total resolution of the lymphadenitis.

The biochemical and drug susceptibility patterns of *M. mageritense* are relatively similar to the formerly known *M. fortuitum* third biovariant complex (*1*,*2*). It is not surprising that they also seem to have the same clinical spectrum (*2*). Although human infections caused by *M. mageritense* are rare, there are case reports involving sinusitis, pneumonia, and hospital-acquired infections, including catheter-related bloodstream infections, implantable cardioverter defibrillator-related infections, prosthetic valve endocarditis, and intrathecal catheter-related meningitis (*2*,*4*,*5*). Skin and soft tissue infections, including parotitis, furunculosis, and surgical site infections, have also been reported (*4*).

Mycobacteria are widespread in nature (*1*) and rapidly growing mycobacteria, such as *M. mageritense*, are ubiquitous in most municipal water supplies (*6*). Although *M. mageritense* has been isolated from cutaneous lesions of a tsunami survivor (*7*) and from 2 patients who received footbaths at the same nail salon (*6*), in most of these case reports, such as for our case, the source of contamination was unknown. Thus, *M. mageritense* is a rapidly growing mycobacteria that can cause granulomatous lymphadenitis in children. Clinicians should be aware of this bacteria during differential diagnoses. 
